# Pivotal Role of AKAP12 in the Regulation of Cellular Adhesion Dynamics: Control of Cytoskeletal Architecture, Cell Migration, and Mitogenic Signaling

**DOI:** 10.1155/2012/529179

**Published:** 2012-06-28

**Authors:** Shin Akakura, Irwin H. Gelman

**Affiliations:** Department of Cancer Genetics, Roswell Park Cancer Institute, Elm and Carlton Streets, Buffalo, NY 14263, USA

## Abstract

Cellular dynamics are controlled by key signaling molecules such as cAMP-dependent protein kinase (PKA) and protein kinase C (PKC). AKAP12/SSeCKS/Gravin (AKAP12) is a scaffold protein for PKA and PKC which controls actin-cytoskeleton reorganization in a spatiotemporal manner. AKAP12 also acts as a tumor suppressor which regulates cell-cycle progression and inhibits Src-mediated oncogenic signaling and cytoskeletal pathways. Reexpression of AKAP12 causes cell flattening, reorganization of the actin cytoskeleton, and the production of normalized focal adhesion structures. Downregulation of AKAP12 induces the formation of thickened, longitudinal stress fibers and the proliferation of adhesion complexes. AKAP12-null mouse embryonic fibroblasts exhibit hyperactivation of PKC, premature cellular senescence, and defects in cytokinesis, relating to the loss of PKC scaffolding activity by AKAP12. AKAP12-null mice exhibit increased cell senescence and increased susceptibility to carcinogen-induced oncogenesis. The paper describes the regulatory and scaffolding functions of AKAP12 and how it regulates cell adhesion, signaling, and oncogenic suppression.

## 1. Introduction

**Figure 1:**
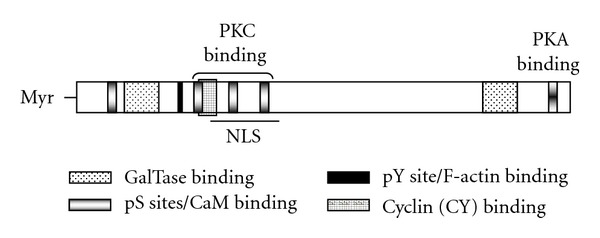
AKAP12 binds to key signaling molecules. AKAP12 contains various demonstrated protein binding domains as well as PKC phosphorylation sites (pS) and a tyrosine phosphorylation site (pY). NLS, nuclear localization signals (at least 4 T_ag_ motifs); CaM, calmodulin; GalTase, *β*1,4-galactosyltransferase; Myr, N-terminal myristoylation.

The actin cytoskeleton plays an essential role in numerous aspects of cell biology such as cell adhesion, cell morphology, cytokinesis, and migration. Cell migration machinery is regulated by signaling intermediates that can be activated by diverse stimuli and that can exert control over a large number of downstream target molecules, all with temporal and spatial specificity [1, 2]. PKA, PKC, and Ca^2+^-binding proteins are examples of cellular regulators that mediate diverse effects on cytoskeletal dynamics, cell adhesion, and cell migration [3, 4]. Control of the subcellular localization of PKA and PKC activities in a temporal manner by A-Kinase-Anchoring Proteins (AKAP) has emerged as a pivotal mechanism to control cell migration [2]. For instance, AKAP12/SSeCKS/Gravin (AKAP12) is thought to control a number of cellular events by scaffolding key signaling molecules such as cyclin D1, calmodulin, PKA, and PKC (Figure 1) [5].

SSeCKS (rodent AKAP12), the Src-Suppressed-C-Kinase-Substrate, was originally identified in a screen for genes severely downregulated by v-Src [6], but subsequently we and others showed that it is also downregulated by oncogenic forms of Ras, Myc and Jun [7, 8] and in SV40-transformed fibroblasts [9]. The gene encoding the human SSeCKS orthologue, Gravin, is localized on chromosome 6q24-25.2, a deletion hotspot in advanced prostate, ovarian, and breast cancer [10], implicating a role for the loss of AKAP12 in cancer progression. Importantly, AKAP12 orthologues have been identified in all vertebrate species, and, in humans and rodents, two major AKAP12 transcripts, *α* and *β*, are expressed ubiquitously in the embryo and the adult as 305- or 290-kDa products (rodents: 290-kDa or 280-kDa), respectively. AKAP12 transcript levels increase in confluent cultures of untransformed cells irrespective of the effects of serum growth factors [7, 10–12], yet, in subconfluent cultures, they are unaffected by either serum deprivation or inhibition of DNA synthesis [13]. Although the coding sequence of AKAP12 contains several PEST motifs linked to protein instability, AKAP12 is a long-lived protein under certain conditions [14]. Newly synthesized AKAP12 in confluent cultures is not well phosphorylated, whereas serum addition to subconfluent cultures results in a rapid serine and tyrosine phosphorylation concurrent with G1 to S progression [13, 15].

AKAP12 is a major in vitro and in vivo substrate of PKC [9, 16]. Both AKAP12 and PKC isozymes (including conventional and novel) contain phosphatidylserine (PS) binding sites, and although PS enhances AKAP12/PKC binding [9, 17], recent data identify two PS-independent PKC binding motifs in AKAP12 [18]. Phosphorylation of AKAP12 in vitro with PKC decreases PS-mediated PKC binding [16], though it has not been shown that this phosphorylation inhibits PS binding itself. Interestingly, PKC-induced phosphorylation of AKAP12 causes it to translocate from plasma membrane and cytoskeletal sites to the perinucleus in fibroblasts, mesangial and epithelial cells [10, 11, 16, 19], suggesting that this event may play a role in the PKC-mediated reorganization of the actin cytoskeleton.

The functions of AKAP12 are based upon its ability to scaffold key signaling proteins in a spatiotemporal manner and specific scaffolding functions have been described for the control of (i) cell migration, (ii) maintenance of cytoskeletal architecture, (iii) cell proliferation, and (iv) cytokinesis.

## 2. Role of AKAP12 on Cellular Architecture, Adhesion, and Migration

One of major scaffolding roles for AKAP12 is as a critical regulator of cell migration. For instance, AKAP12 reexpression is sufficient to inhibit *src*-induced anchorage-independence and Matrigel invasiveness as well as to induce formation of normalized stress fibers and vinculin-associated adhesion plaques typical of those found in untransformed cells [20]. In addition, reexpression of AKAP12 in the rat metastatic prostate cancer cell line, MAT-LyLu, suppresses colony formation in soft agar, decreases refractility, and increases cell-cell interactions [10]. Importantly, AKAP12 attenuates specialized motility, such as chemotaxis and invasiveness, rather than generic cell motility. For instance, AKAP12 reexpression in MAT-LyLu cells has no effect on short- and long-term motility in monolayer wound-healing assays [10]. In contrast, AKAP12 inhibits chemotaxis via the attenuation of a PKC-Raf/MEK/ERK pathway [21]. In addition, upregulated AKAP12 facilitates HGF-induced, c-Met-dependent cell motility through the upregulation of PKA activity and PKA-induced genes, presumably through AKAP12's scaffolding function [22]. Interestingly, AKAP12 phosphorylation by PKC decreases AKAP12-PKC scaffolding but no change in AKAP12-PKA binding [23] or agonist-induced PKA activation [17]. These findings suggest that AKAP12 controls mutually exclusive activation between PKA and PKC. It is likely that the differential PKA-PKC control relates to an overlap between PKC binding and phosphorylation sites mapping to the N-terminus of AKAP12, whereas the PKA binding site maps to the C-terminus of AKAP12 lacking phosphorylation sites (Figure 1). The C-terminal domain of AKAP12 is required for AKAP12 to target PKA to the cell periphery [24]. However, PKC activation by phorbol esters causes translocation of the AKAP12/PKA complex to the perinucleus [23, 24]. Taken altogether, these data strengthen the notion that AKAP12 promotes the differential activation of PKA and PKC in processes such as cell motility.

AKAP12 is involved in FAK-mediated signaling. AKAP12 induces integrin-independent tyrosine phosphorylation of FAK in suspension cultures [25]. AKAP12 reexpression also enhances integrin-mediated adhesion and superinduces FAK autophosphorylation levels, likely by physically disengaging Src from FAK complexes, which, in turn, leads to less focal adhesion turnover (Bing Su, Lingqiu Gao, Fanjie Meng, Li-wu Guo, Julian Rothschild, Irwin H. Gelman, Adhesion-mediated cytoskeletal remodeling is controlled by the direct scaffolding of Src from FAK complexes to lipid rafts by SSeCKS/AKAP12. *Oncogene*, in press). In addition, mitogen-induced, FAK-dependent tyrosine phosphorylation of AKAP12 modulates its binding to the actin-based cytoskeleton, suggesting a role for AKAP12 in mitogen-induced cytoskeletal reorganization [15]. Recently we discovered that AKAP12 negatively regulates FAK expression [26], implicating a role for AKAP12 in the control of FAK levels. Although further work is required to address how AKAP12 controls FAK expression, it is likely that AKAP12 affects both FAK-mediated adhesion and motility pathways.

**Figure 2:**
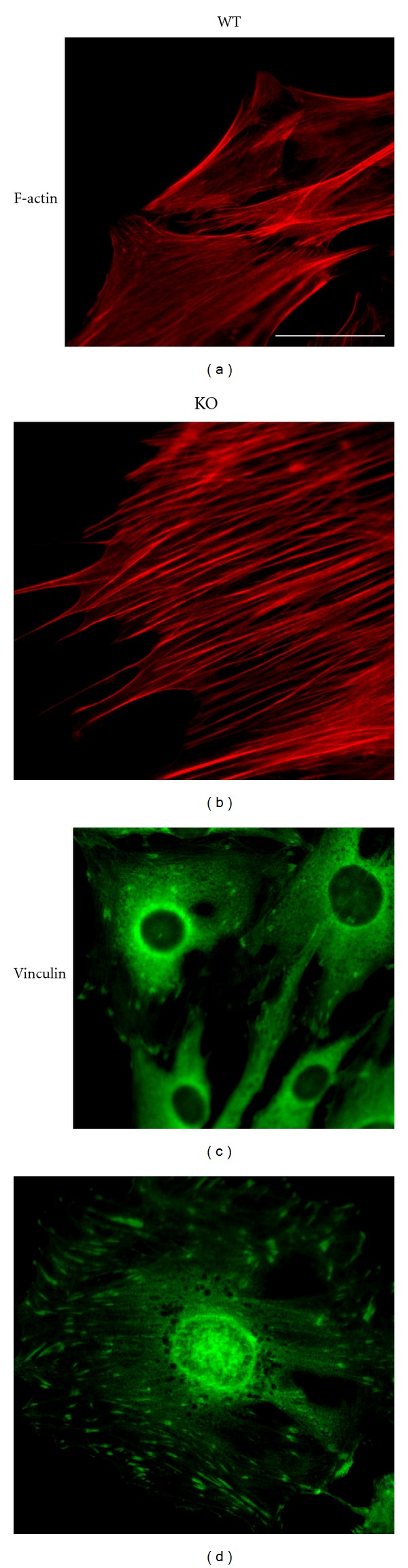
AKAP12-null cells exhibit robust stress fiber formation as well as upregulated focal adhesion complexes. Wild type and AKAP12-null mouse embryonic fibroblasts were stained with either rhodamine-phalloidin (F-actin, (a) and (b)) or antivinculin antibody ((c) and (d)) followed by Alexa488 antibody. Scale bar, 10 *μ*m.

AKAP12 is also a regulator of cytoskeletal architecture. The ablation of AKAP12 expression in glomerular mesangial cells leads to the thickening and polarization of F-actin stress fibers, an increase in the number of transverse focal adhesion plaques, and an increase of phosphotyrosine staining in focal complexes [11]. Consistent with this report, AKAP12-null mouse embryonic fibroblasts (KO-MEF) exhibit robust stress fiber formation as well as increased numbers of focal adhesion plaques ([27], Figure 2). Ablation of AKAP12 converts the stellate morphology of human mesangial cells and rodent fibroblasts to a spindle morphology. In actively dividing cells, AKAP12 associates with a cortical cytoskeleton and is enriched in lamellipodia [16, 25]. Overexpression of AKAP12 in untransformed NIH3T3 cells causes G1-arrest marked by severe cell flattening, elaboration of an AKAP12-associated cytoskeleton, a transient loss of actin stress fibers and vinculin-associated adhesion plaques, and the production of filopodia and lamellipodia-like projections [25, 28]. Ectopic expression of AKAP12 diminishes stress fiber formation likely through a direct binding to F-actin [25] (Figure 1), suggesting that AKAP12 directly affects the formation or maintenance of stress fibers. These observations suggest that AKAP12 plays a critical role in the dynamic reorganization of the actin cytoskeleton during the processes such as cell migration or maintenance of cell architecture.

## 3. Role of AKAP12 on Cell Cycle Regulation and Cytokinesis

Another major scaffolding role for AKAP12 is as a negative regulator of G1 to S progression [6]. It is likely that AKAP12 controls cell cycle progression in NIH3T3 cells by regulating cyclin D1 in two ways: (i) directly scaffolding cyclin D1 via cyclin D1 binding (CY) motifs, thereby facilitating contact-inhibition by sequestering cyclin D1 pools in the cytoplasm, and (ii) decreasing cyclin D1 expression by suppressing serum-inducible ERK2 activity [28]. The CY domains overlap with major PKC phosphorylation sites on AKAP12 (Figure 1). The notion that AKAP12 scaffolding activity for cyclin D1 is antagonized after it is phosphorylated by PKC comes from the findings that (i) activation of PKC by phorbol esters induces cyclin D1 translocation to the nucleus, and (ii) phosphorylation of AKAP12 by PKC in vitro antagonizes its binding to cyclin D1 and cyclin E [29].

Intriguingly, AKAP12 changes its localization during the cell cycle. Consistent with previous reports [16, 23, 24], live cell imaging analysis demonstrates that localization of AKAP12 of resting cells is mainly in the cytoplasm, with some portion localizing to specific compartments such as plasma membrane and perinucleus (S. Akakura and I. H. Gelman, unpublished observation). Although further work is required to elucidate the role of AKAP12 in cell cycle regulation, it is conceivable that AKAP12 localization and the timing of expression are critical for cell cycle progression.

Evidence is accumulating that AKAP12 is involved in cytokinesis regulation [30–32]. We [27] and others [30, 32] suggested that the loss of AKAP12 leads to cytokinesis defects. A recent systems biology analysis implicates the existence of a mitotic protein complex containing AKAP12 and other molecules such as Polo-like kinase 4, APC, dynein and profilin (http://www.mitocheck.org/cgi-bin/mtc?query=MCG_0000007). Choi et al. demonstrated that AKAP12 localizes on the anaphase abscission furrow [30]. The furrow contains the actin-myosin ring whose PKC-Rho GTPase-dependent contraction helps complete daughter chromosome separation [33], and, thus, it is conceivable that AKAP12 regulates cytokinesis via its ability to scaffold PKC and F-actin, and to attenuate Rho GTPase activity [34]. Since AKAP12 controls the activity of PKC, and PKC*ε* mediates the completion of cytokinesis [35], it is conceivable that AKAP12 normally scaffolds PKC and regulates actomyosin ring formation through controlling PKC*ε*-RhoA signaling during specific mitotic stages and structures.

## 4. AKAP12 as a Tumor Suppressor and Metastasis Suppressor

Despite the fact that AKAP12 is widely expressed throughout embryogenesis [14], AKAP12-null mice (KO-mice) are viable though they exhibit spontaneous prostatic hyperplasia [36]. Dysplastic foci were observed less frequently but were associated with the loss of E-cadherin staining and the loss of basal cell markers [36], suggesting that the loss of AKAP12 causes a cancer-prone condition. In fact, prostates of KO mice exhibit senescence associated *β*-galactosidase expression [27], which can be used as a premalignant marker [37], implicating that the loss of AKAP12 causes a precancerous condition. KO-MEF also exhibit premature senescence marked by senescence-associated *β*-galactosidase expression. These cells are readily transformed by single oncogenes such as Src or Ras, suggesting that the loss of AKAP12's tumor suppressor function renders the cell transformation-prone. Importantly, AKAP12 deficiency causes hyperactivation of PKC isozymes, leading to Rb-dependent senescence involving PKC*α* and *δ* but not PKC*ε*. KO-MEF are also more susceptible to immortalization in culture [27]. Immortalized KO-MEF have decreased levels of senescence associated *β*-galactosidase staining and of the cyclin-kinase inhibitor, p16, indicating that the cells override Rb-dependent senescence. Moreover, expression levels of Cyclin-dependent kinase 4 and LATS/Warts (a mitotic kinase), which are downregulated in KO-MEF, are upregulated in immortalized KO-MEF. These data suggest that AKAP12 facilitates pathways for continued proliferation through G1 and G2 phase arrest points found in senescent cells.

It is likely that AKAP12 controls senescence through a direct scaffolding of PKC isozymes because reexpression of full-length AKAP12, but not AKAP12 deleted of its PKC-binding domains, suppresses senescence [27]. This suggests that AKAP12-null cells would be a unique tool to study the biological effects of PKC isozyme hyperactivation.

Rhim et al. reported that the protein levels of AKAP12 are higher in senescent human diploid fibroblasts and in aging rat and human keratinocytes [38]. Given our finding that KO-MEF express higher levels of p47phox, a component of NADPH oxidase, it is reasonable to speculate that AKAP12 suppresses the production of reactive oxygen species (ROS), ultimately serving as a protection against premature aging and spontaneous oncogenesis. Further work is required to address how AKAP12 regulates the aging process.

Evidence is accumulating that AKAP12 expression is downregulated in many cancer types, either associated with gene deletion or epigenetic downregulation due to promoter hypermethylation or changes in chromatinization. For instance, the expression level of AKAP12 is downregulated in breast cancer [39], leukemia [40], ovarian cancer [41], colorectal cancer [42], and hepatocellular carcinoma [43]. Many microarray-based studies demonstrate significant reduction in relative AKAP12 mRNA levels in many cancer types that cited in Entrez GEO (Gene Expression Omnibus) or Oncomine (http://www.oncomine.org/) linking AKAP12 expression with tumor suppression. In addition, we showed recently that AKAP12-null mice have increased susceptibility to papilloma and squamous cell carcinoma formation induced by DMBA and TPA, well-known skin carcinogens [26]. Interestingly, dermal layers in AKAP12-null mice are hyperplastic, and they show significant upregulation of FAK, a known promoter of carcinogen-induced squamous cell carcinoma [44].

AKAP12 has been shown to function as a metastasis suppressor possibly by inhibiting the expression of VEGF at distal sites [45], and by inhibiting oncogenic invasiveness [21]. Reexpression of AKAP12 in MAT-LyLu cells causes a small decrease in primary subcutaneous tumor growth yet severely suppresses the formation of macroscopic lung metastasis [10]. In addition, multiple Oncomine studies show significant decreases in AKAP12 expression in metastases compared to levels in primary tumors, suggesting a role for AKAP12 in suppressing metastasis. Taken altogether, these data suggest that AKAP12 is especially potent in regulating the metastatic process, a function likely relating to its ability to downregulate angiogenesis-controlling genes, such as VEGF [46], and invasion-controlling genes, such as MMP-2 [21].

## 5. Other Roles for AKAP12

There is mounting evidence that AKAP12 scaffolding of PKC plays a role in regulation of mesangial cell differentiation and proliferation, and thus, glomerular function [11]. Nelson et al. reported that AKAP12 mediates the control of the actin-based cytoskeletal architecture in mesangial cells by PKC [11]. Recently, Burnworth et al. reported that SSeCKS controls the localization and activity of cyclin D1 in glomerular parietal epithelial cells and influences response to proliferative injury in the glomerulus [47]. This paper demonstrates severely increased proliferative injury levels of glomerular parietal epithelial cells, leading to proteinurea in AKAP12-null versus wild type mice. Thus, AKAP12 plays a critical role in architectural maintenance of glomerular parietal epithelial cells, and AKAP12 deficiency increases the susceptibility to injury-induced glomerulonephritis [47].

AKAP12 is involved in the *β*_2_-adrenergic receptor-mediated signaling [48]. Agonist stimulation of the *β*_2_-adrenergic receptor leads to activation of kinases that are associated with AKAP12. PKA-mediated phosphorylation of AKAP12 stabilizes the interaction between AKAP12 and the receptor, while PKC-mediated phosphorylation of AKAP12 causes it to translocate from the *β*_2_-adrenergic receptor. Prolonged agonist stimulation leads to degradation of the receptor and induces desensitization [48, 49].

AKAP12 is also critical in the regulation of blood-brain barrier (BBB) [45, 50]. AKAP12 attenuates neovascularization as well as barrier formation through the downregulation of proangiogenic genes such as HIF1*α* or VEGF [46, 50]. AKAP12 is upregulated during normoxic transition of the mouse embryo at birth, and AKAP12 is responsible for suppressing brain angiogenesis through a JNK-dependent downregulation of VEGF and for inducing postnatal formation of the BBB by promoting tighter astrocyte/endothelial cell junctions [50]. Recently, Kwon et al. reported that AKAP12 is essential for the integrity of the endothelium by maintaining the expression of PAK2 and AF6, cell-cell adhesion regulators, during vascular development [51]. Although AKAP12-null mice do not exhibit blood vessel issues [36], deficiency of AKAP12 causes hemorrhage in embryos of zebrafish and overexpression of PAK2 and AF6 is sufficient to rescue the abnormal hemorrhage in AKAP12-depleted zebrafish embryos. Taken altogether, AKAP12 is essential for cellular architecture and is required for the integrity of cell-cell junction.

## 6. Conclusion

AKAP12 regulates cell cycle progression, cell motility, and cell morphology through its multiple scaffolding domains. Suppression of oncogenic proliferation, chemotaxis, and cellular senescence all involve attenuation of PKC activation through direct spatiotemporal scaffolding functions of AKAP12.

 Several major issues regarding AKAP12 remain to be elucidated. First, relating to its subcellular localization, it is still not clear whether AKAP12 plays a role in the nucleus, even though it contains at least six nuclear localization signals. Second, the molecular mechanisms by which AKAP12 differentially regulates the crosstalk between PKA and PKC signaling pathways remain unclear. Lastly, other AKAP12 binding partners are likely to be found that either regulate AKAP12 functions or that are regulated by AKAP12 scaffolding.

 Further studies are required to elucidate how the regulation of PKC and other molecules through scaffolding proteins such as AKAP12 maintains the integrity of cellular signaling and cytoskeletal control. Results from those studies would strongly suggest that targeting PKC-regulators such as AKAP12 should have therapeutic benefit for cancer patients.

## Abbreviations

AKAP12: A-kinase-anchoring protein 12
SSeCKS: Src-suppressed C-kinase substrate
PKA: Cyclic AMP-dependent protein kinase
PKC: Protein kinase C
MEF: Mouse embryonic fibroblasts.
